# Reconstructing protein structure from solvent exposure using tabu search

**DOI:** 10.1186/1748-7188-1-20

**Published:** 2006-10-27

**Authors:** Martin Paluszewski, Thomas Hamelryck, Pawel Winter

**Affiliations:** 1 Department of Computer Science, University of Copenhagen, Universitetsparken 1, 2100 Copenhagen, Denmark; 2 Bioinformatics Center, Institute of Molecular Biology, University of Copenhagen, Universitetsparken 15 building 10, 2100 Copenhagen, Denmark

## Abstract

**Background:**

A new, promising solvent exposure measure, called *half-sphere-exposure *(HSE), has recently been proposed. Here, we study the reconstruction of a protein's *C*_*α *_trace solely from structure-derived HSE information. This problem is of relevance for *de novo *structure prediction using predicted HSE measure. For comparison, we also consider the well-established contact number (CN) measure. We define energy functions based on the HSE- or CN-vectors and minimize them using two conformational search heuristics: *Monte Carlo simulation *(MCS) and *tabu search *(TS). While MCS has been the dominant conformational search heuristic in literature, TS has been applied only a few times. To discretize the conformational space, we use lattice models with various complexity.

**Results:**

The proposed TS heuristic with a novel tabu definition generally performs better than MCS for this problem. Our experiments show that, at least for small proteins (up to 35 amino acids), it is possible to reconstruct the protein backbone solely from the HSE or CN information. In general, the HSE measure leads to better models than the CN measure, as judged by the RMSD and the angle correlation with the native structure. The angle correlation, a measure of structural similarity, evaluates whether equivalent residues in two structures have the same general orientation. Our results indicate that the HSE measure is potentially very useful to represent solvent exposure in protein structure prediction, design and simulation.

## Background

**Figure 1:**
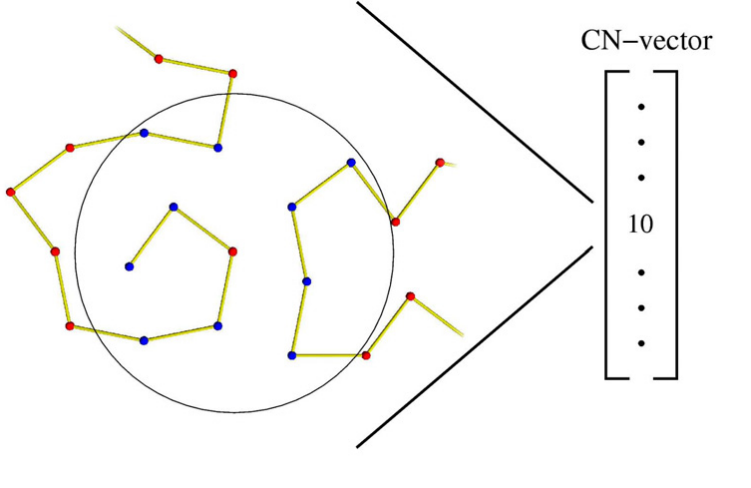
**CN**. The contact number (CN) of a residue.

The extent to which an amino acid in a protein is accessible to the surrounding solvent is highly dependent on the type of amino acid. In general, hydrophilic amino acids tend to be near the solvent accessible surface, while hydrophobic amino acids tend to be buried in the core of the protein. To measure this effect, several solvent exposure measures have been proposed [1-7], and one of these is the *contact number measure *(CN) [7]. The CN of a residue is the number of *C*_*α *_atoms in a sphere centered at the *C*_*α *_atom of the residue in question (Figure 1). The CN of all residues of a protein is called the *CN vector*. The CN vector is well conserved and can be predicted with high accuracy [8].

**Figure 2:**
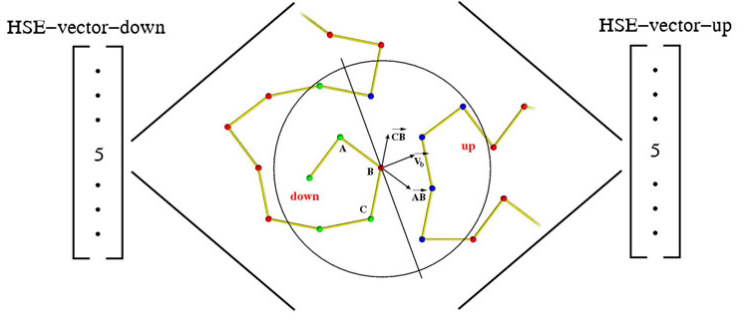
**HSE**. Given the positions of 3 consecutive *C*_*α *_atoms (*A*, *B*, *C*), the approximate side-chain direction V→
 MathType@MTEF@5@5@+=feaafiart1ev1aaatCvAUfKttLearuWrP9MDH5MBPbIqV92AaeXatLxBI9gBaebbnrfifHhDYfgasaacH8akY=wiFfYdH8Gipec8Eeeu0xXdbba9frFj0=OqFfea0dXdd9vqai=hGuQ8kuc9pgc9s8qqaq=dirpe0xb9q8qiLsFr0=vr0=vr0dc8meaabaqaciaacaGaaeqabaqabeGadaaakeaacuWGwbGvgaWcaaaa@2DF3@_*b *_can be computed as the sum of AB→
 MathType@MTEF@5@5@+=feaafiart1ev1aaatCvAUfKttLearuWrP9MDH5MBPbIqV92AaeXatLxBI9gBaebbnrfifHhDYfgasaacH8akY=wiFfYdH8Gipec8Eeeu0xXdbba9frFj0=OqFfea0dXdd9vqai=hGuQ8kuc9pgc9s8qqaq=dirpe0xb9q8qiLsFr0=vr0=vr0dc8meaabaqaciaacaGaaeqabaqabeGadaaakeaadaWhcaqaaiabdgeabjabdkeacbGaay51Gaaaaa@3078@ and CB→
 MathType@MTEF@5@5@+=feaafiart1ev1aaatCvAUfKttLearuWrP9MDH5MBPbIqV92AaeXatLxBI9gBaebbnrfifHhDYfgasaacH8akY=wiFfYdH8Gipec8Eeeu0xXdbba9frFj0=OqFfea0dXdd9vqai=hGuQ8kuc9pgc9s8qqaq=dirpe0xb9q8qiLsFr0=vr0=vr0dc8meaabaqaciaacaGaaeqabaqabeGadaaakeaadaWhcaqaaiabdoeadjabdkeacbGaay51Gaaaaa@307C@. The plane perpendicular to V→
 MathType@MTEF@5@5@+=feaafiart1ev1aaatCvAUfKttLearuWrP9MDH5MBPbIqV92AaeXatLxBI9gBaebbnrfifHhDYfgasaacH8akY=wiFfYdH8Gipec8Eeeu0xXdbba9frFj0=OqFfea0dXdd9vqai=hGuQ8kuc9pgc9s8qqaq=dirpe0xb9q8qiLsFr0=vr0=vr0dc8meaabaqaciaacaGaaeqabaqabeGadaaakeaacuWGwbGvgaWcaaaa@2DF3@_*b *_cuts the sphere centered at *B *in an upper and a lower hemisphere.

Recently, a new promising solvent exposure measure, called *half-sphere-exposure *(HSE), has been proposed [9]. While the CN measure uses a single sphere centered at the *C*_*α *_atom, the HSE measure considers two hemispheres. Two values, an *up *and a *down *value, are associated with each residue, corresponding to the upper and lower hemisphere. The geometry of the HSE construction is shown schematically in Figure 2. The up and down HSE values measure two fundamentally different environments of an amino acid, one of them corresponding to the neighbourhood of the side chain [9]. The HSE measure compares favorably with other solvent exposure measures in terms of computational complexity, sensitivity, correlation with the stability of mutants and conservation. An important advantage of the HSE measure is that it can be calculated from *C*_*α *_-only or other simplified protein models. Therefore, it forms an attractive alternative to the use of the CN measure in protein structure prediction methods [10].

Here, we study if it is possible to reconstruct a protein's *C*_*α *_trace solely from a CN vector or an HSE vector. These vectors are obtained from the protein's known native state and our goal is therefore to evaluate the information contents of these measures. This problem could become important for *de novo *structure prediction, for example if predicted HSE values are used as restraints. Preliminary results show that the HSE measure can be predicted with reasonably high accuracy[11]. Reconstruction of a protein structure from a predicted HSE vector might thus be an attractive way of approaching the sequence-to-structure problem. Predicted CN-/HSE vectors are expected to have errors compared to the exact vectors. The results presented in this paper are based on exact vectors and therefore provide an upper bound on the information contents of predicted CN-/HSE vectors. If protein structure prediction was carried out on a *predicted *HSE vector only, it is expected that the results would not be better than the results presented in this paper. It would therefore be natural to add other predictable information such as secondary structure, radius of gyration etc. to a structure prediction system using predicted HSE vectors. The problem of reconstructing protein structure from vectors of one-dimensional structural information has been studied before. Kinjo et al.[12] used exact vectors of *secondary structure *(SS), CN and *residue-wise contact order *(RWCO) together with refinement using the AMBER force field to reconstruct native like structures. Their results show that SS and CN information without the use of RWCO is not enough to reconstruct native like structures. Unfortunately, prediction methods for the RWCO measure only have moderate performance as compared to SS and CN[12].

Porto et al.[13] described an algorithm for reconstructing the contact map (CM) from its principal (one-dimensional) eigenvector. However, methods for predicting a high quality eigenvector are not likely to exist. Here, we only consider measures that potentially can be predicted with high accuracy. Furthermore we only use one type of measure (either CN or HSE), which is important for evaluating the information content of a measure. To this end, we compare structure reconstruction using an energy function based on the HSE measure with an energy function that uses the well-established CN measure.

If an approximate CN-/HSE vector is obtained from a prediction method, there might be no structure that exactly realizes the vector. In that case, we are interested in finding a structure with a CN- or HSE-vector *similar *to the predicted vector. Therefore we define energy functions based on the HSE- or CN-vectors and minimize them using two conformational search heuristics: *Monte Carlo simulation *(MCS) and *tabu search *(TS). MCS has been widely used for protein structure prediction, and TS has been applied with great success to many optimization problems, but has rarely been used for protein structure prediction [14-16].

In this article, the radius of the HSE sphere is chosen to be 12 Å for all experiments. The *optimal *radius has yet to be determined, both in terms of predictability and reconstructability. If the radius is too small, important residue pairs might be overlooked. On the other hand, if the radius is too large, many irrelevant residues are considered. In this respect, 12 Å seems to be a good compromise [9].

The rest of the article is organized as follows. In the next section we describe the energy function based on the HSE measure. Then the protein abstraction and lattice model are discussed. In section *Heuristics*, we present the two conformational search heuristics, MCS and TS. In section *Lattice experiments*, MCS and TS are evaluated in lattices of different complexity. Finally, we evaluate the information content (that is, to what extent they can be used to reconstruct a protein structure) of the HSE and CN measures using TS and a high complexity lattice.

### HSE energy function

The similarity of two HSE vectors A and B of length N can be measured using the following RMS deviation:

RMSD(A,B)=∑i=1N((Aui−Bui)2+(Adi−Bdi)2)2N,
 MathType@MTEF@5@5@+=feaafiart1ev1aaatCvAUfKttLearuWrP9MDH5MBPbIqV92AaeXatLxBI9gBaebbnrfifHhDYfgasaacH8akY=wiFfYdH8Gipec8Eeeu0xXdbba9frFj0=OqFfea0dXdd9vqai=hGuQ8kuc9pgc9s8qqaq=dirpe0xb9q8qiLsFr0=vr0=vr0dc8meaabaqaciaacaGaaeqabaqabeGadaaakeaacqqGsbGucqqGnbqtcqqGtbWucqqGebarcqGGOaakcqWGbbqqcqGGSaalcqWGcbGqcqGGPaqkcqGH9aqpdaGcaaqaamaalaaabaWaaabmaeaacqGGOaakcqGGOaakcqWGbbqqdaWgaaWcbaGaemyDau3aaSbaaWqaaiabdMgaPbqabaaaleqaaOGaeyOeI0IaemOqai0aaSbaaSqaaiabdwha1naaBaaameaacqWGPbqAaeqaaaWcbeaakiabcMcaPmaaCaaaleqabaGaeGOmaidaaOGaey4kaSIaeiikaGIaemyqae0aaSbaaSqaaiabdsgaKnaaBaaameaacqWGPbqAaeqaaaWcbeaakiabgkHiTiabdkeacnaaBaaaleaacqWGKbazdaWgaaadbaGaemyAaKgabeaaaSqabaGccqGGPaqkdaahaaWcbeqaaiabikdaYaaakiabcMcaPaWcbaGaemyAaKMaeyypa0JaeGymaedabaGaemOta4eaniabggHiLdaakeaacqaIYaGmcqWGobGtaaaaleqaaOGaeiilaWcaaa@5B92@

where {A,B}ui
 MathType@MTEF@5@5@+=feaafiart1ev1aaatCvAUfKttLearuWrP9MDH5MBPbIqV92AaeXatLxBI9gBaebbnrfifHhDYfgasaacH8akY=wiFfYdH8Gipec8Eeeu0xXdbba9frFj0=OqFfea0dXdd9vqai=hGuQ8kuc9pgc9s8qqaq=dirpe0xb9q8qiLsFr0=vr0=vr0dc8meaabaqaciaacaGaaeqabaqabeGadaaakeaacqGG7bWEcqWGbbqqcqGGSaalcqWGcbGqcqGG9bqFdaWgaaWcbaGaemyDau3aaSbaaWqaaiabdMgaPbqabaaaleqaaaaa@35D6@ and {A,B}di
 MathType@MTEF@5@5@+=feaafiart1ev1aaatCvAUfKttLearuWrP9MDH5MBPbIqV92AaeXatLxBI9gBaebbnrfifHhDYfgasaacH8akY=wiFfYdH8Gipec8Eeeu0xXdbba9frFj0=OqFfea0dXdd9vqai=hGuQ8kuc9pgc9s8qqaq=dirpe0xb9q8qiLsFr0=vr0=vr0dc8meaabaqaciaacaGaaeqabaqabeGadaaakeaacqGG7bWEcqWGbbqqcqGGSaalcqWGcbGqcqGG9bqFdaWgaaWcbaGaemizaq2aaSbaaWqaaiabdMgaPbqabaaaleqaaaaa@35B4@ are the up and down values of the i'th index. RMSD (*A*, *B*) can be used to describe the *energy *of structure *S*_*A *_where A is the HSE vector of *S*_*A *_and B is the HSE vector of the native structure. These definitions are easily extended to the CN measure. The energy functions are the only optimization criteria used by the MCS and TS algorithms.

### The protein model

**Figure 3:**
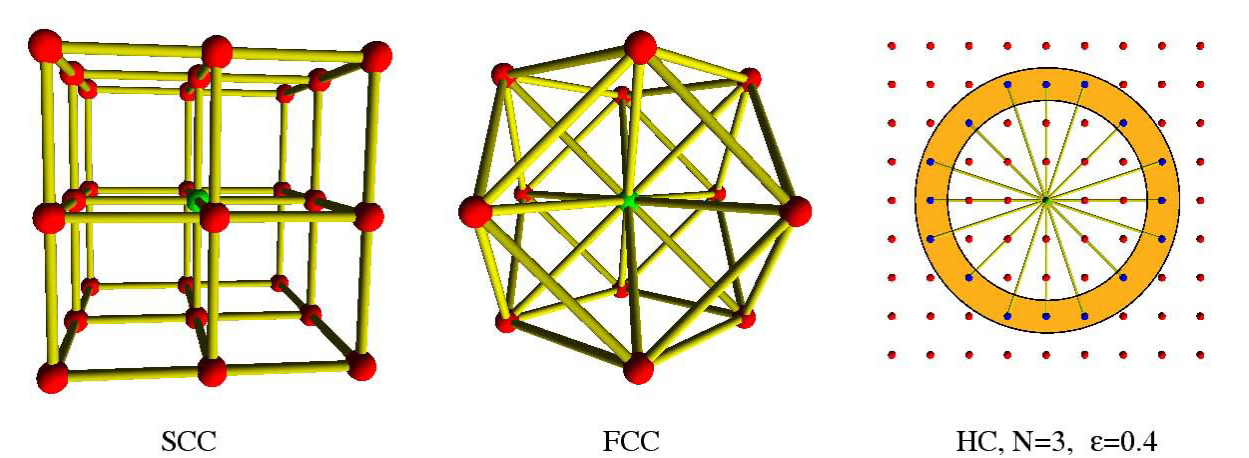
**Lattices**. Interior nodes of the SCC and FCC lattices are connected to respectively 6 and 12 neighbouring nodes. Nodes of high coordination lattices have many neighbours because of variable edge size.

The HSE and CN energy functions only depend on the positions of the *C*_*α *_atoms in the protein backbone. This allows us to simplify the problem by considering a protein as a chain of connected points representing the positions of the *C*_*α *_atoms. Furthermore, to reduce and discretize the conformational space of the protein, we require the *C*_*α *_atoms of the chain to be positioned on a 3D lattice. A lattice can be defined as a set of basis vectors corresponding to the directions to the neighbouring nodes. The basis vectors of the *simple cubic lattice *(SCC) are the cyclic permutations of [± 1,0,0] ([1,0,0], [-1,0,0], [0,1,0], [0,-1,0], [0,0,1], [0,0,-1]) and the basis vectors of the *face centered cubic lattice *(FCC) are the cyclic permutations of [± 1, ± 1,0] ([1,1,0], [1,0,1], [1,-1,0], [1,0,-1], [-1,1,0], [-1,0,1], [-1,-1,0], [-1,0,-1], [0,1,1], [0,1,-1], [0,-1,1], [0,-1,-1]). This gives 6 basis vectors for SCC and 12 for FCC as illustrated in Figure 3. The length of an edge between two neighbouring nodes is taken to be 3.8 Å which is the average distance between two consecutive *C*_*α *_atoms in proteins.

Lattice models are widely used for studying the fundamental properties of protein structure[17]. Such models have for example provided invaluable insights on topics such as the validity of pairwise energy functions[18], the evolution of protein superfamilies[19] and the importance of local structural bias in the determination of a protein's fold[20]. Many lattice models have been proposed and evaluated in the literature. Not surprisingly, experiments show a high correlation between the number of basis vectors of a lattice and its ability to represent a protein backbone[21,22]. When deciding on a lattice model, one must always consider the trade-off between the reduction of the conformational space and the quality of the structure representation. Therefore, in section *Lattice experiments *we evaluate four different lattices of various complexity: The SCC lattice, the FCC lattice and two *high coordination *(HC) lattices with 54 and 390 basis vectors, respectively.

A high coordination lattice has an underlying cubic lattice with unit length less than 3.8/*N *Å for some integer *N *> 1. Cubic lattice points are connected in the high coordination lattice if their Euclidean distance is between 3.8 ± *ε *for some *ε *> 0. The high coordination lattices used here are named HC4 and HC8 corresponding to their N value (4 and 8). The *ε *value is 0.2 for all HC lattices. Figure 3 shows an illustration of a 2D high coordination lattice with *N *= 3 and *ε *= 0.4. High coordination lattices have previously been used for protein structure prediction[23,24]. Note that the SCC and FCC lattices both have the excluded volume property, meaning that atoms at two different lattice points will never collide. This property does not necessarily hold for high coordination lattices, and collisions must therefore be detected explicitly.

### Heuristics

We apply two iterative search heuristics for minimization of the HSE energy. One of them is the tabu search *metaheuristic *proposed by F. Glover in 1989[25,26]. A metaheuristic is a general framework that can be specialized to solve various optimization problems. For many problems in Operations Research (OR), tabu search is the metaheuristic of choice. However, for protein structure prediction, tabu search has only been given a modest amount of attention[14-16].

**Figure 4:**
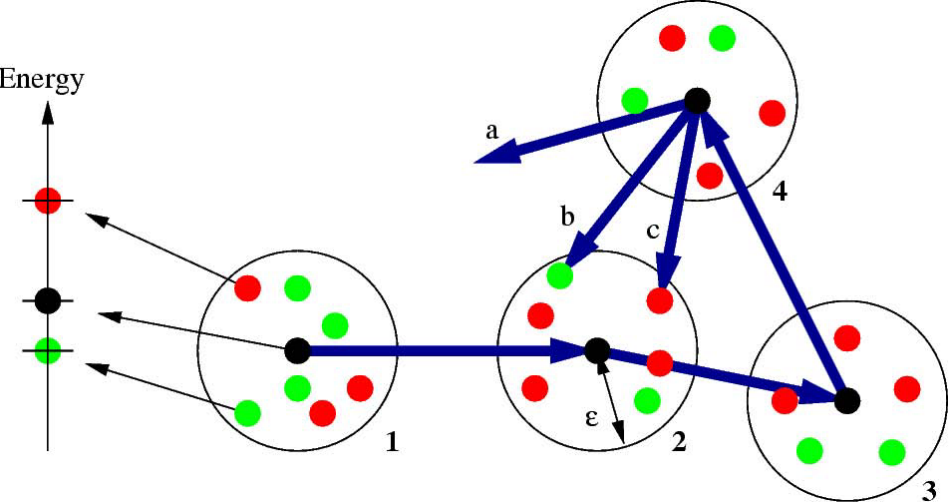
**Explicit- and implicit tabu structures**. Black points represent explicit tabu structures and red points represent implicit tabu structures.

**Figure 5:**
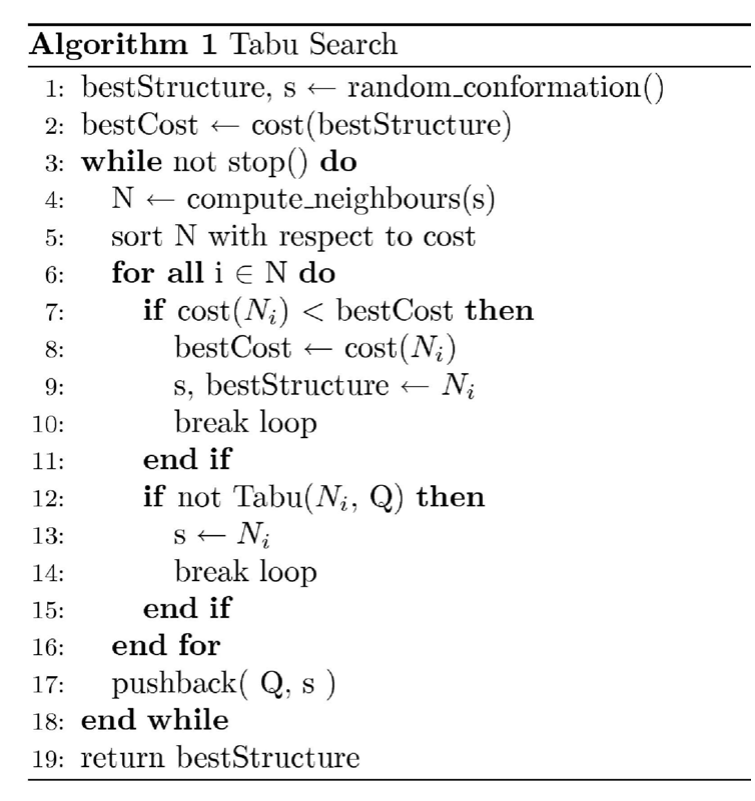
Algorithm 1.

**Figure 6:**
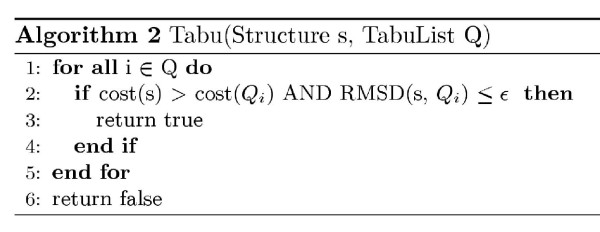
Algorithm 2.

In Algorithm 1 and 2 (Figures 5 and 6) the pseudo code for tabu search is shown. TS is basically a local improvement heuristic where the best structure in a neighbourhood is repeatedly selected. However, memory is used to prevent cycling in local minima. A previous TS implementation [16] inserts visited structures into a *tabu list *and only consider new structures if they are not in the tabu list. We have found that extending the tabu definition improves the performance considerably. Here, we still keep a list of previously visited structures in a so-called *explicit tabu list*. Each structure in the explicit tabu list defines a set of *implicit tabu structures*. Given a structure *E *in the explicit tabu list, a structure *I *is said to be implicit tabu if the distance-RMSD (dRMSD) between *E *and *I *is less than *ε *and the energy of *I *is greater than or equal to the energy of *E*. The adjustable parameter *ε *is called the *tabu difference*. Figure 4 illustrates a sequence of visited structures (black points) in a solution space. Only the visited structures are inserted in the explicit tabu list. The additional green and red points correspond to structures within *ε *dRMSD of the explicit tabu structures. Green points are structures with lower energy and red points are structures with higher energy than the explicit tabu structure. When choosing a new solution in the neighbourhood three things can happen, *a) *A solution is more than *ε *dRMSD away from all explicit tabu structure. *b) *the solution is within *ε *dRMSD, and the energy is *lower *than the explicit tabu structure, *c) *the solution is within *ε *dRMSD, and the energy is *higher *than the explicit tabu structure. Structures that comply with case *c *are said to be *implicit tabu *and cannot be visited. Note that when *ε *= 0 the search heuristic works as a regular TS heuristic since only visited structures become tabu. The use of implicit tabu structures is new in the context of protein structure prediction. However, in TS implementations for OR problems it is a common technique to make features of a solution tabu, such that regions of the search space become tabu.

We have also applied standard Monte Carlo simulation (MCS) for minimizing the HSE energy. MCS heuristics are stochastic and therefore differ from TS by being nondetermistic. An MCS iteration consists of randomly choosing a protein conformation in the neighbourhood of a current conformation. For a fixed temperature T, the new protein conformation is accepted with the probability

*p *= *e*^-Δ*E*/*T*^,

where ΔE is the difference between the energy of the current conformation and the new conformation. A protein conformation is modelled as a list of N vectors, where N is the number of *C*_*α *_atoms of the protein. The neighbourhood of both MCS and TS consists of conformations resulting from changes of one, two or three consecutive indices. A single index change results in a new structure where one part of the structure is fixed and the other part is translated. Two or three indices are changed locally such that the parts of the structure before and after the changing indices are fixed. All local index changes between two lattice points can be stored in a table to speed up the computation time significantly.

### Lattice experiments

**Figure 7:**
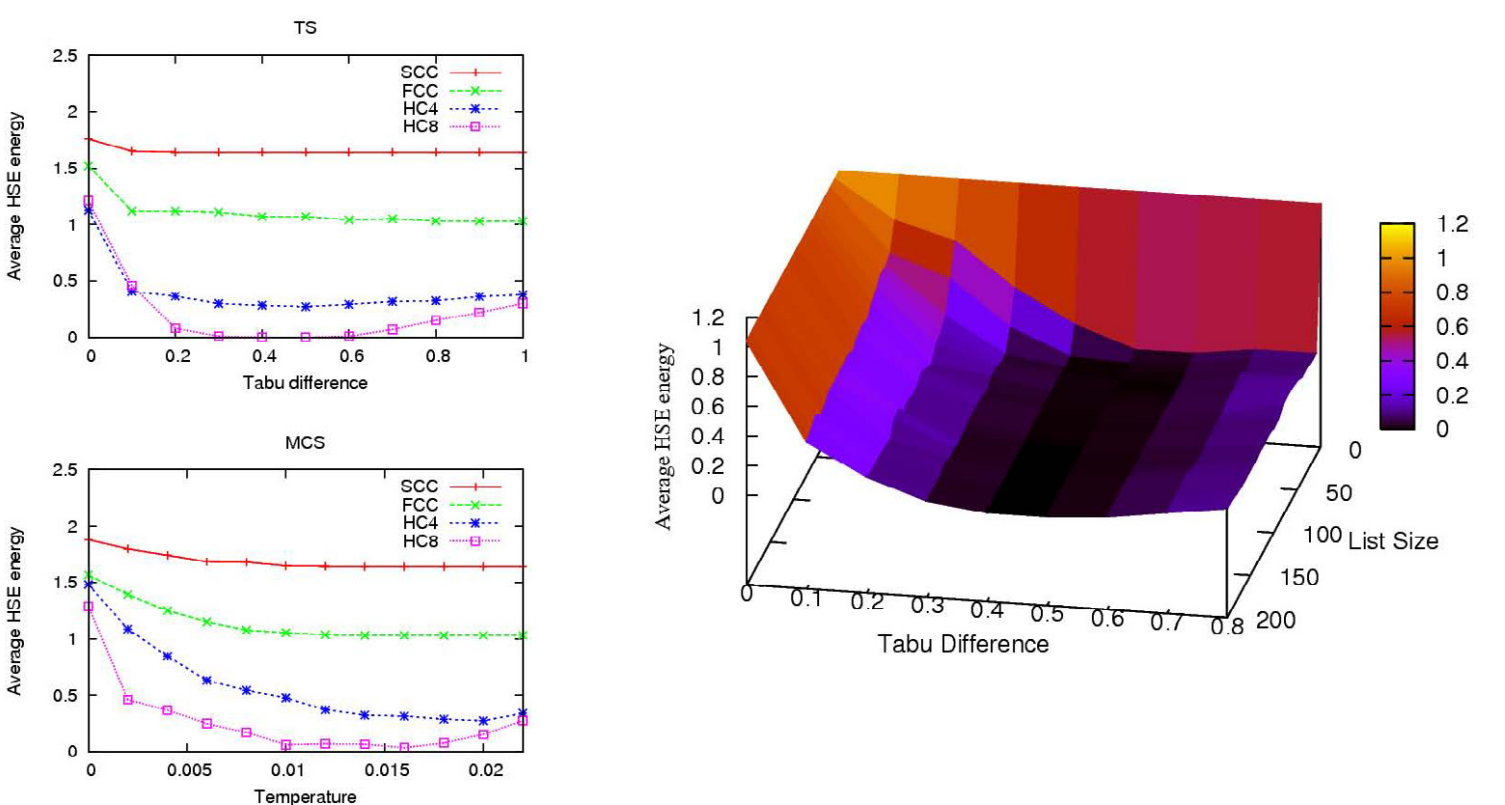
**Lattice experiments**. The two first plots show the values in table 1 and 2. The right figure shows the average HSE energy on HC8 with variable tabu list size and variable tabu difference.

Here, we evaluate TS and MCS on lattices of different complexity. The purpose of the experiments in this section is to tune the parameters (lattice type, tabu difference, temperature). In the next section we fix the parameters to their optimal values found here and compare the HSE and CN measures on different proteins. For each lattice, the heuristics are initialized with 20 random conformations using different parameter values. The variable parameter of MCS is the temperature and the variable parameter of TS is the tabu difference. Each run is stopped after 15 minutes and the structure with the lowest observed HSE energy is reported. To get reasonable running times for these experiments, the HSE energy is based on the native structure of the small protein *Protegrin 1 *(1PG1, 18 residues). Tables 1 and 2 show the results of the lattice experiments for the TS and MCS heuristics. There is a row for each lattice type and data columns show the average HSE energy found over the 20 runs for the various parameters. In the SCC lattice, structures with the same HSE energy are found in all 20 runs (tabu difference 0.4 and 0.5), but the best observed HSE energy is rather high. The reason is that the SCC lattice is very coarse grained and low energy structures therefore do not exist in this lattice. For lattices of increasing complexity, the ability to find structures with lower energy increases. TS and MCS seem to perform equally well in low complexity lattices. However, in high coordination lattices, the TS heuristic performs slightly better than MCS on average. For the lattice with highest complexity (HC8) TS found zero energy structures for all 20 runs, this robustness was not observed for the MCS heuristic. These results indicate that conformational search heuristics using the HSE measure require high complexity lattices or off-lattice models with a high degree of freedom. Furthermore, TS is slightly more robust that MCS in high coordination lattices. The results of experiments with variable tabu list size and variable tabu difference in the HC8 lattice are shown in Figure 7. The figure shows that the tabu list size should generally be more than 50 elements, and there is no gain of having a very long list.

**Table 1:** Average HSE energy for *Protegrin 1 *using *TS *on various lattices and tabu differences.

	Tabu difference										
Lattice	0.0	0.1	0.2	0.3	0.4	0.5	0.6	0.7	0.8	0.9	1.0
SCC	1.76	1.65	**1.64**	**1.64**	**1.64**	**1.64**	**1.64**	**1.64**	**1.64**	**1.64**	**1.64**
FCC	1.52	1.12	1.12	1.11	1.07	1.07	1.04	1.05	**1.03**	**1.03**	**1.03**
HC4	1.13	0.41	0.36	0.30	0.28	**0.27**	0.29	0.32	0.32	0.36	0.38
HC8	1.21	0.46	0.08	0.01	**0.00**	**0.00**	0.01	0.07	0.15	0.22	0.30

The best averages for each lattice type are boldfaced. The column with 0.0 tabu difference corresponds to the results of a regular TS implementation with no implicit tabu structures.

**Table 2:** Average HSE energy for *Protegrin 1 *using *MCS *on various lattices and temperatures.

	Temperature											
Lattice	0.000	0.002	0.004	0.006	0.008	0.010	0.012	0.014	0.016	0.018	0.020	0.022
SCC	1.88	1.80	1.74	1.68	1.68	1.65	**1.64**	**1.64**	**1.64**	**1.64**	**1.64**	**1.64**
FCC	1.57	1.39	1.25	1.15	1.08	1.05	**1.03**	**1.03**	**1.03**	**1.03**	**1.03**	**1.03**
HC4	1.48	1.09	0.85	0.63	0.54	0.48	0.37	0.32	0.31	0.29	**0.27**	0.34
HC8	1.29	0.46	0.37	0.25	0.17	0.06	0.07	0.07	**0.04**	0.08	0.15	0.28

The best averages for each lattice type are boldfaced.

### Comparison of HSE and CN measures

In the previous section, experiments on a small protein show that minimization of the HSE energy in high coordination lattices leads to structures with HSE vectors that are very similar (or equal) to the native structure. In this section, experiments on proteins of varying size are done using the TS heuristic with tabu difference 0.4 and the HC8 lattice. The energy functions are based on the HSE vectors of native structures as described in section *HSE energy function*. In addition to the HSE energy, the CN energy is considered for comparison. The main purpose of the experiments is to examine the reconstructability of a protein's backbone solely from the information stored in the HSE-/CN vectors.

Each TS run is started from a random structure which is iteratively improved as described in section *Heuristics*. For these experiments we want to start TS on 100 random structures that are as different from each other as possible. Therefore, to effectively sample the search space, 10000 random conformations are initially generated. Ideally, from this set of 10000 conformations, we would like to choose the set of 100 conformations such that the minimum RMSD between any two conformations is maximized. This problem is generally known as the p-dispersion problem and is NP hard[27]. Solving this problem to optimality is therefore not feasible, so we use a greedy heuristic to find a good set of 100 different random conformations. The greedy heuristic works by first picking a random conformation. The following 99 conformations are then picked one at a time, such the minimimum RMSD to any of the already picked conformations is maximized.

For each protein, the energy function based on its native structure is minimized for each of the 100 random starting conformations and the structures with lowest energy are reported. The search is stopped after 12 hours or if the energy reaches zero. Zero energy means that a structure with exactly the same HSE- or CN vector as the native structure is found (but not necessarily identical structures).

To evaluate the quality of the structures with low energy, the RMSD with the native structure and *angle correlation *[28,29] is used. Angle correlation is a measure with the following definition. For each *C*_*α*_, let V→
 MathType@MTEF@5@5@+=feaafiart1ev1aaatCvAUfKttLearuWrP9MDH5MBPbIqV92AaeXatLxBI9gBaebbnrfifHhDYfgasaacH8akY=wiFfYdH8Gipec8Eeeu0xXdbba9frFj0=OqFfea0dXdd9vqai=hGuQ8kuc9pgc9s8qqaq=dirpe0xb9q8qiLsFr0=vr0=vr0dc8meaabaqaciaacaGaaeqabaqabeGadaaakeaacuWGwbGvgaWcaaaa@2DF3@_*α *_be the vector pointing in the side chain direction (see Figure 2). Let Vαmc→
 MathType@MTEF@5@5@+=feaafiart1ev1aaatCvAUfKttLearuWrP9MDH5MBPbIqV92AaeXatLxBI9gBaebbnrfifHhDYfgasaacH8akY=wiFfYdH8Gipec8Eeeu0xXdbba9frFj0=OqFfea0dXdd9vqai=hGuQ8kuc9pgc9s8qqaq=dirpe0xb9q8qiLsFr0=vr0=vr0dc8meaabaqaciaacaGaaeqabaqabeGadaaakeaacqWGwbGvdaqhaaWcbaacciGae8xSdeMaemyBa0Maem4yamgabaGaeyOKH4kaaaaa@3453@ be the vector pointing in the direction of the mass center, and let *θ*_*α *_be the angle between V→
 MathType@MTEF@5@5@+=feaafiart1ev1aaatCvAUfKttLearuWrP9MDH5MBPbIqV92AaeXatLxBI9gBaebbnrfifHhDYfgasaacH8akY=wiFfYdH8Gipec8Eeeu0xXdbba9frFj0=OqFfea0dXdd9vqai=hGuQ8kuc9pgc9s8qqaq=dirpe0xb9q8qiLsFr0=vr0=vr0dc8meaabaqaciaacaGaaeqabaqabeGadaaakeaacuWGwbGvgaWcaaaa@2DF3@_*α *_and Vαmc→
 MathType@MTEF@5@5@+=feaafiart1ev1aaatCvAUfKttLearuWrP9MDH5MBPbIqV92AaeXatLxBI9gBaebbnrfifHhDYfgasaacH8akY=wiFfYdH8Gipec8Eeeu0xXdbba9frFj0=OqFfea0dXdd9vqai=hGuQ8kuc9pgc9s8qqaq=dirpe0xb9q8qiLsFr0=vr0=vr0dc8meaabaqaciaacaGaaeqabaqabeGadaaakeaacqWGwbGvdaqhaaWcbaacciGae8xSdeMaemyBa0Maem4yamgabaGaeyOKH4kaaaaa@3453@. The angle correlation measure is the average of the differences in *θ*_*α *_between the optimized structure and the native structure. Zero angle correlation is perfect correlation, 90° is random correlation and 180° is perfect 'anti'-correlation. Note that the CN- and HSE vectors of a structure are identical to the vectors of the mirror of the structure. Therefore, in the following results, if the RMSD between a structure and its native mirror image is smaller we report this value instead. All computations were performed on a 236 nodes Dell Optiplex GX260 cluster (2,4 GHz P4, 512 Mb RAM).

## Results and discussion

**Figure 8:**
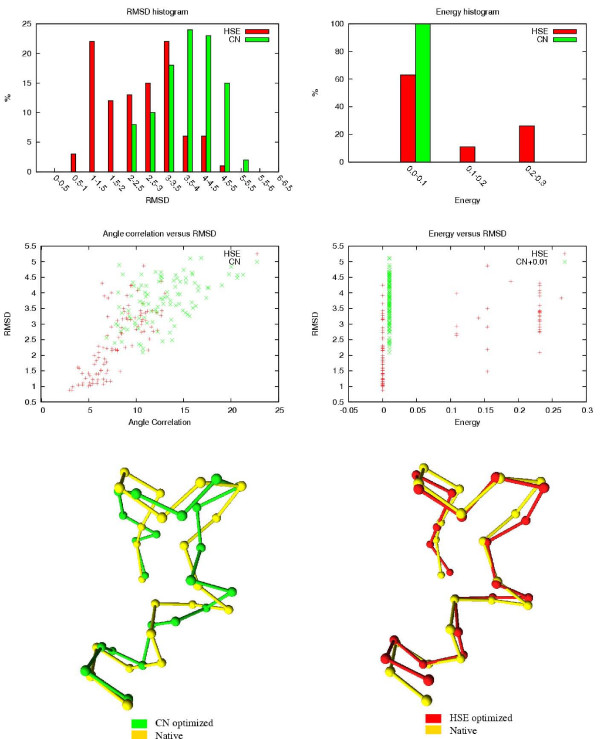
**Human Endothelin (1EDN), 21 residues**. In the energy versus RMSD plot, the CN values have an offset of 0.01 for better illustration.

**Figure 9:**
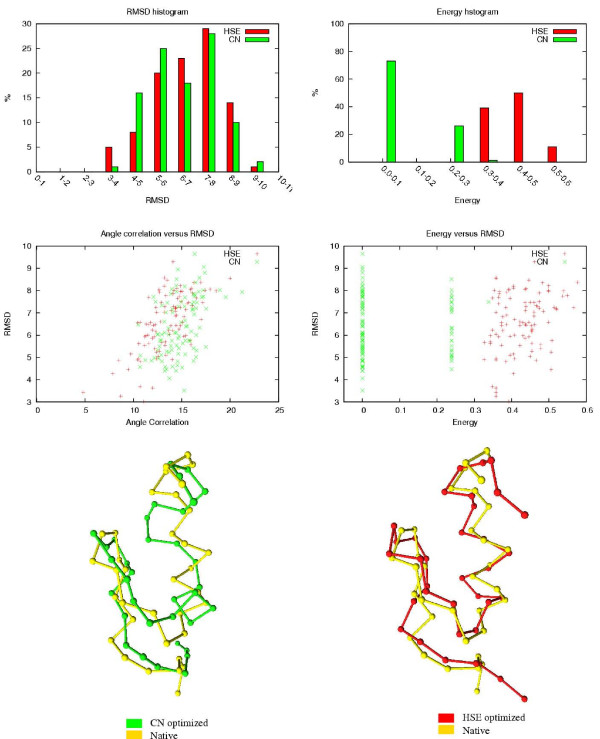
Third Zinc Finger (1SRK). 35 residues.

**Figure 10:**
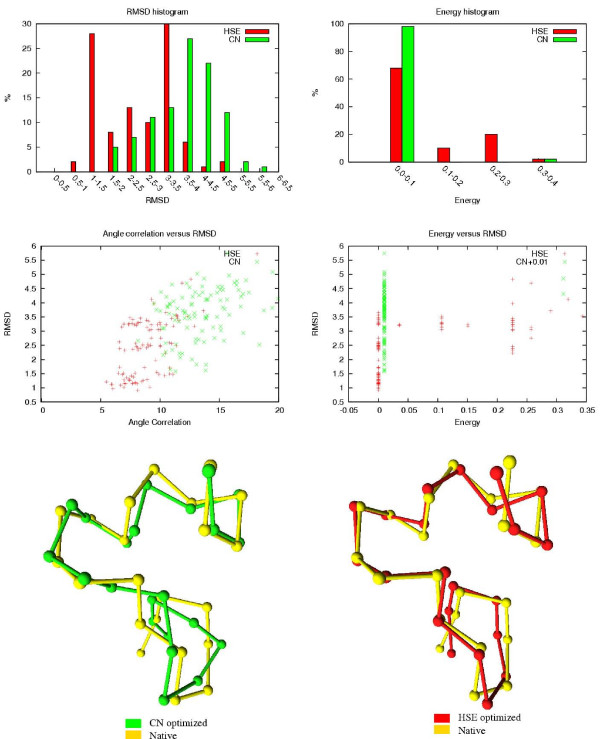
**Mu-Conotoxin GIIA (1TCH). 23 residues**. In the energy versus RMSD plot, the CN values have an offset of 0.01 for better illustration.

**Figure 11:**
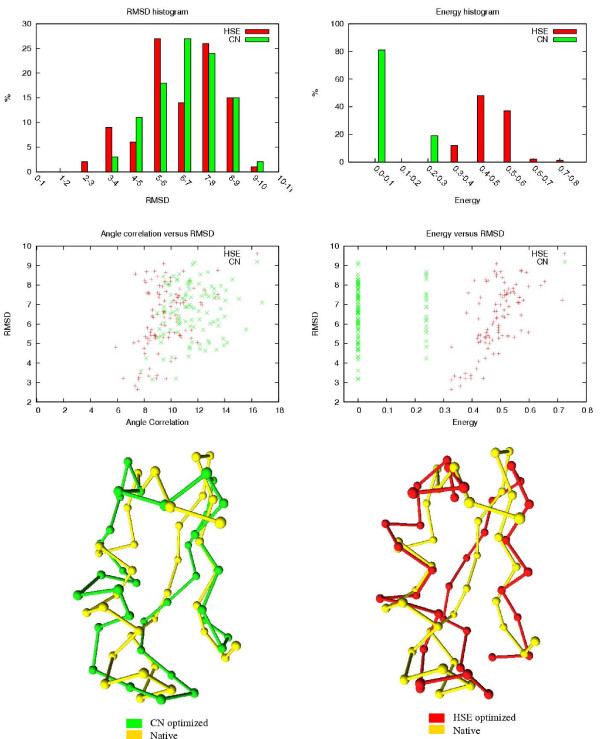
Pandinus Toxin (2PTA). 35 residues.

**Figure 12:**
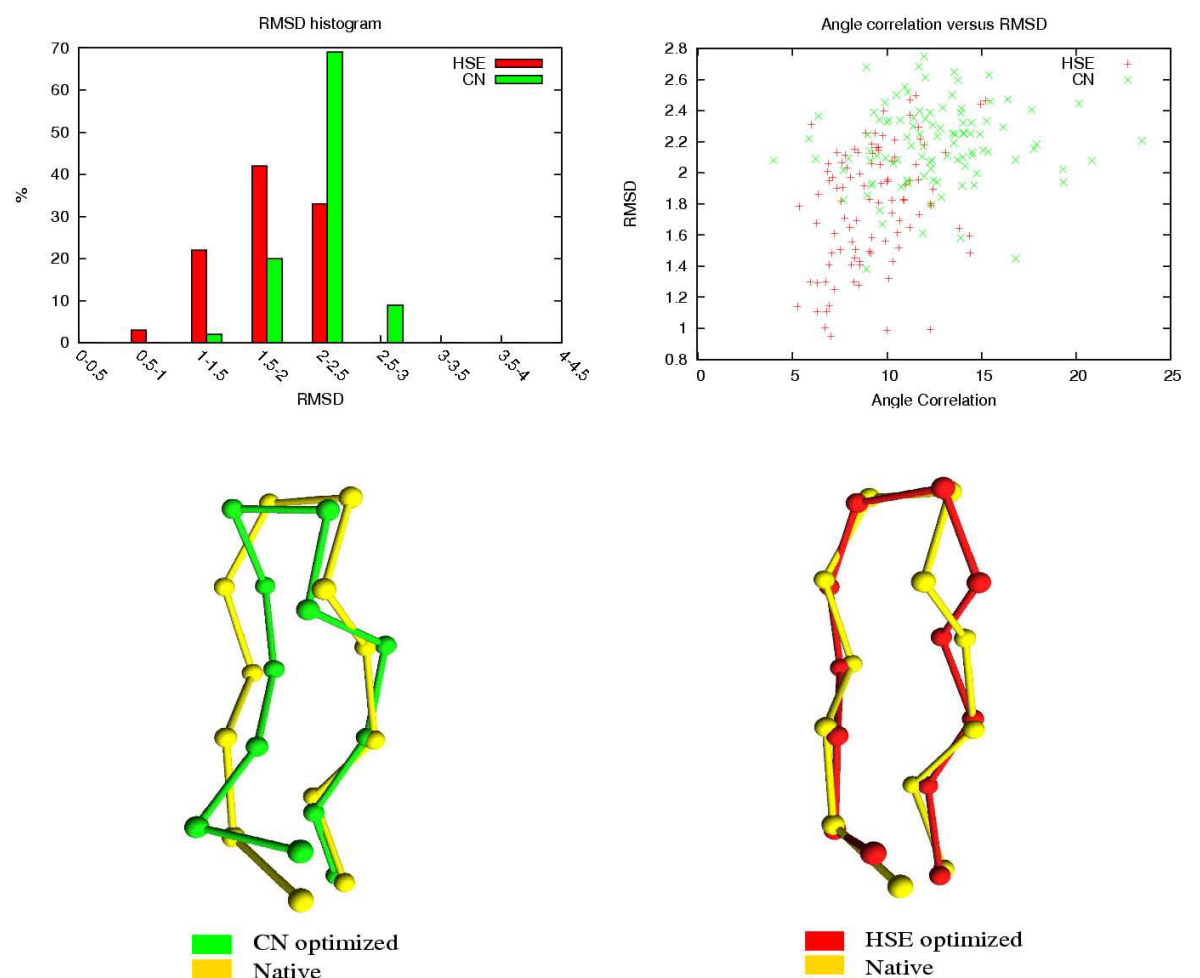
**Tryptophan Zipper 1 (1LEO). 13 residues**. All optimized structures have zero energy.

The results of the HSE and CN comparisons are shown in Table 3. The table shows how many of the 100 HSE/CN minimized conformations are below a certain RMSD threshold. The associated RMSDs and energy values of the 100 conformations are also shown. In Figures 8 to 12, histograms show the RMSD and energy distribution of the CN- or HSE-optimized structures. The histograms reveal that most of the lowest energy structures are similar to the native structure. This trend is much more prevalent for the HSE-optimized structures. Based on the histograms, we conclude that the CN-/HSE-energy functions have a large smooth minimum around the structure of the native state and few smaller local minima scattered around the conformational space.

**Table 3:** Comparison of the HSE- and CN measures for various proteins.

Residues	Measure	< 7 Å RMSD	< 6 Å RMSD	< 5 Å RMSD	< 4 Å RMSD	< 3 Å RMSD	< 2 Å RMSD	lowest RMSD	lowest energy
		*Human Endothelin *(1EDN)							
21	CN	100	100	98	60	18	0	**2.09**	**0.00**
	HSE	100	100	100	93	65	37	**0.88**	**0.00**

		*Tryptophan Zipper 1*(1LE0)							
13	CN	100	100	100	100	100	22	**1.38**	**0.00**
	HSE	100	100	100	100	100	67	**0.95**	**0.00**

		*Third Zinc Finger *(1SRK)							
35	CN	60	42	17	(1SRK) 1	0	0	**3.52**	**0.00**
	HSE	56	33	13	5	0	0	**3.02**	**0.33**

		*Mu-Conotoxin GIIA *(1TCH)							
23	CN	100	100	97	63	23	5	**1.58**	**0.00**
	HSE	100	100	100	97	61	38	**0.91**	**0.00**

		*Pandinus Toxin *(2PTA)							
35	CN	59	32	14	3	0	0	**3.17**	**0.00**
	HSE	58	44	17	11	2	0	**2.66**	**0.33**

Scatter plots show the *angle correlation *vs. RMSD. The Figures also show the best HSE- and CN-optimized structures superimposed on the native structure. The *yellow *backbone is the native structure, the *red *backbone is the best HSE optimized structure and the *green *backbone is the best CN optimized structure.

The CN and HSE comparisons show that low HSE-energy structures are generally closer to the native structure than low CN-energy structures, this both in terms of RMSD and angle correlation. A backbone structure with a good angle correlation implies that the general orientation of the residues is accurate. The plots show that this property is much more prevalent in HSE-optimized structures. Existing protein structure prediction methods that use the CN measure could therefore benefit from using the HSE measure instead of the CN measure.

Here we have developed a lattice model for protein structure prediction using the CN-/HSE energy functions. The search heuristic is based on TS with a novel tabu definition and the results indicate that TS performs better than MCS for this problem. TS with this new tabu definition might also be applied with success for other protein structure optimization problems.

Lattice experiments suggest that near zero energy structures only exists in high coordination lattices. Therefore, when using the HSE measure the model should have a high degree of freedom. All results are found using small proteins (the largest protein has 35 amino acids). When using larger proteins, it becomes very time consuming to find low energy structures and they are often not native like.

We have shown that it is possible to reconstruct the backbone of small proteins using the HSE vector of the native structure. Obviously, a predicted HSE vector would have some errors or noise as compared to the exact HSE vector. A future research project could therefore be to analyze the reconstructability of a protein backbone using HSE vectors with various degree of noise. Other directions could be to consider a more detailed energy function using other predictable information such as secondary structure. Another option could be to enforce protein-like geometry, using for example angular constraints.

In this article, we only considered lattice models. However, off-lattice models and other conformational search heuristics such as replica exchange MCMC[30] could be considered as well.
